# Genotyping and pathobiologic characterization of canine parvovirus circulating in Nanjing, China

**DOI:** 10.1186/1743-422X-10-272

**Published:** 2013-08-29

**Authors:** Yanbing Zhao, Yan Lin, Xujian Zeng, Chengping Lu, Jiafa Hou

**Affiliations:** 1 College of Veterinary Medicine, Nanjing Agricultural University, Nanjing, China

**Keywords:** Canine parvovirus, Genetic analysis, Dog infection

## Abstract

**Background:**

Canine parvovirus (CPV) is an important pathogen that causes acute enteric disease in dogs. It has mutated and spread throughout the world in dog populations. We provide an update on the molecular characterization of CPV that circulated in Nanjing, a provincial capital in China between 2009 and 2012.

**Results:**

Seventy rectal swab samples were collected from the dogs diagnosed with CPV infection in 8 animal hospitals of Nanjing. Sequence analysis of VP2 genes of 31 samples revealed that 29 viral strains belonged to CPV-2a subtype, while other two strains were classified into CPV-2b. To investigate the pathogenicity of the prevalent virus, we isolated CPV-2a and performed the animal experiment. Nine beagles were inoculated with 10^5.86^ of 50% tissue culture infectious doses (TCID_50_) of the virus. All the experimentally infected beagles exhibited mild to moderate mucoid or watery diarrhea on day 4 post-infection (p.i.). On day 9 p.i., characteristic histopathological lesions were clearly observed in multiple organs of infected dogs, including liver, spleen, kidney, brain and all segments of the small and large intestines, while viral DNA and antigen staining could be detected in the sampled tissues. It is notable that canine parvovirus was isolated in one from two brain samples processed.

**Conclusion:**

Our results indicated that CPV-2a is the predominant subtype in Nanjing of China. And this virus caused extensive lesions in a variety of tissues, including the brain.

## Background

The canine parvovirus (CPV) infection is a viral illness that most commonly affects puppies. The infected dogs develop an acute gastroenteritis characterized by loss of appetite, vomiting, fever, diarrhea (from mucoid to haemorrhagic) and leucopenia [1]. CPV, a member of the *Parvovirus* genus, contains a single strand DNA genome of about 5200 nucleotides that is packaged in an icosahedral capsid [2]. There are three capsid proteins, VP1, VP2 and VP3. VP2 is the major capsid protein, and it plays an important role in determining viral host ranges and tissue tropisms [3]. Amino acids substitutions in VP2 gene have been responsible for genetic and antigenic properties [4]. The virus emerged as dog pathogen in the late 1970's as host variant of feline panleukopenia virus (FPV) [5,6]. A few years after its emergence, the original virus type CPV-2 was replaced by two new antigenic variants, CPV-2a and CPV-2b [7,8]. Recently, a novel CPV mutant, CPV-2c, is widely distributed and co-existing with other CPV types in Europe [9,10], North [11,12] and South America [13,14] countries.

In China, CPV infections were first observed as sporadic cases during 1982 (unpublished data). Subsequently, widespread outbreaks of canine hemorrhagic enteritis with high morbidity and mortality occurred over the whole country [15]. Along with the increasing number of pet dogs in China, CPV infection has emerged as a veterinary public health concern that affect puppies because of its high mortality and morbidity [16]. However there has been too little information concerning the antigenic types of CPV prevailing in China.

The identification of the subtypes of CPV-2 that are currently circulating in the canine population is essential for the understanding of viral evolution and the development of measures to control its spread [17]. Antigenic differences have been demonstrated between CPV variants in neutralisation tests [18]. There is concern that the antigenic differences may decrease the effectiveness of the vaccine based on the original antigenic type, CPV-2. Although the original vaccine has been shown to protect dogs against challenge from any of the current CPV types [19], there are still many cases of clinical parvovirus in dogs. For example, in 2007, an outbreak of CPV-2c was reported in Italy in the vaccinated dogs with CPV-2-based vaccine [20]. Some authors have suggested an update of the virus strains in current vaccines, taking into account the existing partial protection [21,22]. Considering this, it is important for us to isolate new parvovirus variants circulating in the field in order that more effective vaccines are prepared from an immunogenic point of view.

To provide an update on the molecular characterization of CPV that circulated in Nanjing, China, in the study, we characterized canine parvovirus from fecal samples of domestic dogs by polymerase chain reaction followed by sequencing, isolated the prevalent virus and performed *in vivo* experiments to investigate the pathogenicity of this virus.

## Results

### Prevalence and genetic characterization of canine parvovirus

One DNA band of the expected size (583 bp), corresponding to the partial amplification of the VP2 gene, was observed by gel electrophoresis in all the 70 samples diagnosed with CPV infection. Thirty-one samples were selected for sequence analysis. Then the sequences were submitted to GenBank. GenBank accession numbers of the VP2 genes of 31 samples are numbered from KC556927 to KC556957. Sequence comparisons showed nucleotide identities of 98.2–100% among the CPV strains in Nanjing, China. Nucleotide sequences were translated into aa sequences to identify the types. Only two (designated CPV-JS60 and CPV-JS63) out of the 31 samples presented a GAT codon at the same position of VP2 protein, characteristic of CPV-2b, while other 29 samples presented an AAT codon at position 426 of the VP2 protein, characteristic of CPV-2a. The main differences in some amino acids of the CPV VP2 gene products are summarized in Table 1. Specifically, these strains had Asp or His at position 427, Thr or Ala at position 440, Thr or Asn at position 445, Pro or His at position 512, and Pro or Thr at position 580.

**Table 1:** The main differences in some amino acids of the VP2 gene of CPV strains analyzed in this study

**Strains**	**Origin**	**Year**	**Accession no.**	**Place of amino acid site**												**Gene type**
				**426**	**427**	**436**	**440**	**445**	**450**	**474**	**476**	**512**	**515**	**577**	**580**	
*Reference strains*																
CPV-15	U.S.	1984	M24003	N	D	I	T	T	T	F	T	P	S	Q	P	CPV-2a
CPV-V120	Vietnam	2000	AB054215	N	D	I	T	T	T	F	T	P	S	Q	P	CPV-2a
CPV-39	U.S.	1984	M74849	D	D	I	T	T	T	F	T	P	S	Q	P	CPV-2b
CPV-Taichung	Taiwan	2004	AY869724	D	D	I	T	T	T	F	T	P	S	Q	P	CPV-2b
CPV-G7/97	Germany	1997	FJ005196	E	D	I	T	T	T	F	T	P	S	Q	P	CPV-2c
*Chinese strains*																
CPV-JS1	Nanjing	2009	KC556927	N	H	-	-	N	-	-	-	-	-	-	-	CPV-2a
CPV-JS2	Nanjing	2009	KC556928	N	H	F	A	N	-	-	-	-	-	-	-	CPV-2a
CPV-JS3	Nanjing	2009	KC556929	N	H	-	A	N	-	-	-	-	-	-	-	CPV-2a
CPV-JS4	Nanjing	2009	KC556930	N	H	-	-	-	-	-	-	-	-	-	-	CPV-2a
CPV-JS11	Nanjing	2010	KC556931	N	-	-	-	N	-	-	-	-	-	-	-	CPV-2a
CPV-JS12	Nanjing	2010	KC556932	N	-	-	A	-	-	-	-	-	-	-	-	CPV-2a
CPV-JS13	Nanjing	2010	KC556933	N	-	-	A	-	-	-	-	-	-	-	T	CPV-2a
CPV-JS14	Nanjing	2010	KC556934	N	-	-	-	-	-	-	-	-	-	-	T	CPV-2a
CPV-JS15	Nanjing	2010	KC556935	N	-	-	-	-	-	-	-	-	-	-	T	CPV-2a
CPV-JS16	Nanjing	2010	KC556936	N	-	-	A	-	-	-	-	-	-	-	-	CPV-2a
CPV-JS17	Nanjing	2010	KC556937	N	-	-	A	-	-	-	-	-	-	-	-	CPV-2a
CPV-JS18	Nanjing	2010	KC556938	N	-	-	-	N	-	-	-	H	-	-	-	CPV-2a
CPV-JS38	Nanjing	2011	KC556939	N	-	-	A	-	-	-	-	-	-	-	-	CPV-2a
CPV-JS39	Nanjing	2011	KC556940	N	-	-	A	-	-	-	-	-	-	-	-	CPV-2a
CPV-JS40	Nanjing	2011	KC556941	N	-	-	A	N	-	-	-	-	F	H	T	CPV-2a
CPV-JS41	Nanjing	2011	KC556942	N	H	-	-	N	N	-	-	H	-	-	T	CPV-2a
CPV-JS42	Nanjing	2011	KC556943	N	H	-	A	N	-	-	-	H	-	-	-	CPV-2a
CPV-JS43	Nanjing	2011	KC556944	N	-	-	-	-	-	S	-	-	-	-	T	CPV-2a
CPV-JS44	Nanjing	2011	KC556945	N	-	-	-	-	-	-	-	-	-	-	-	CPV-2a
CPV-JS45	Nanjing	2011	KC556946	N	H	-	A	N	N	-	-	H	F	-	-	CPV-2a
CPV-JS46	Nanjing	2011	KC556947	N	-	-	-	N	-	-	I	-	-	-	-	CPV-2a
CPV-JS47	Nanjing	2011	KC556948	N	-	-	-	-	-	-	-	-	-	-	-	CPV-2a
CPV-JS58	Nanjing	2012	KC556949	N	-	-	-	-	-	-	-	-	-	-	-	CPV-2a
CPV-JS59	Nanjing	2012	KC556950	N	-	-	A	-	-	-	-	-	-	-	-	CPV-2a
CPV-JS60	Nanjing	2012	KC556956	D	-	-	-	-	-	-	-	-	-	-	-	CPV-2b
CPV-JS61	Nanjing	2012	KC556951	N	-	-	-	-	-	-	-	-	-	-	-	CPV-2a
CPV-JS62	Nanjing	2012	KC556952	N	-	-	A	-	-	-	-	-	-	-	-	CPV-2a
CPV-JS63	Nanjing	2012	KC556957	D	-	-	A	-	-	-	-	-	-	-	-	CPV-2b
CPV-JS64	Nanjing	2012	KC556953	N	-	-	A	-	-	-	-	-	-	-	-	CPV-2a
CPV-JS65	Nanjing	2012	KC556954	N	-	-	-	-	-	-	-	-	-	-	-	CPV-2a
CPV-JS66	Nanjing	2012	KC556955	N	-	-	A	-	-	-	-	-	-	-	-	CPV-2a

Identical amino acids are represented by -.

### Virus isolation and experimental infection

Considering that CPV-2a is epidemiologically predominant in dog populations of Nanjing in China, we isolated the type CPV-2a from three fecal samples in order to further investigate its pathogenicity. Out of three samples, one isolate, CPV-JS2, was obtained. After second blind passages, typical CPE in the form of rounding, increased granularity and detached cells appeared in the infected F81 cells. The observation of electron microscope showed a large quantity of 20–25 nm virus particles with cubic symmetry, typical of parvovirus (data not shown). The titre of the isolated virus determined by the method of Reed and Munch was 10^4.86^ TCID_50_/100 μl.

**Figure 1:**
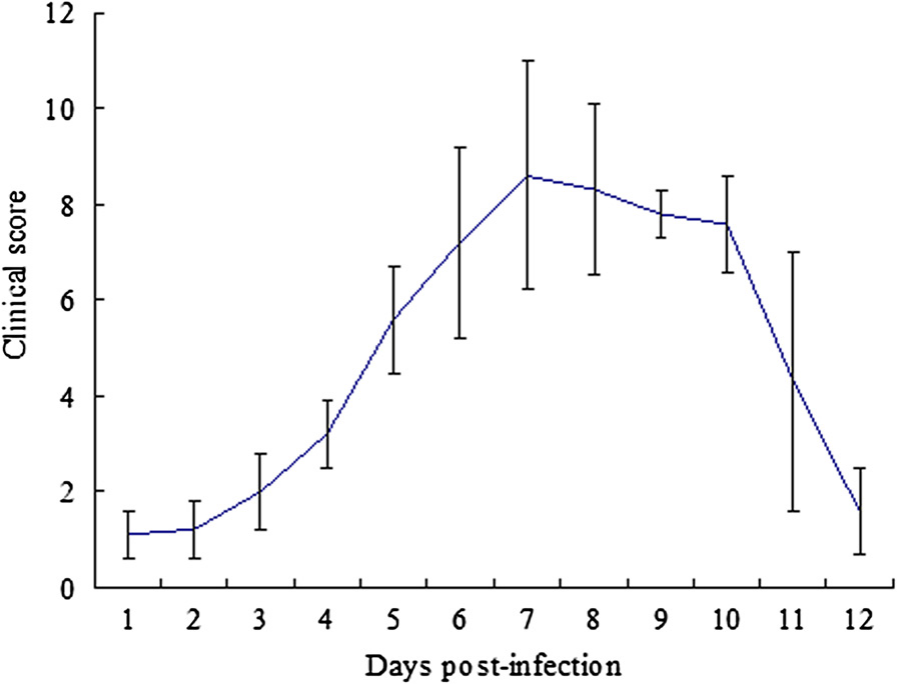
**Clinical scores of dogs infected experimentally with CPV-JS2.** The data are expressed as the mean ± standard error.

For investigating the pathogenicity of the prevalent virus, we performed the artificial infection experiment of CPV-JS2 in beagles. Clinical signs in the dogs given the isolated virus orally were exhibited on day 3 post-infection (p.i.), when four out of nine dogs developed fever (body temperature ≥38.9°C), following anorexia, depression, and mild dehydration. Mild to moderate mucoid or watery diarrhea were exhibited in all dogs of experimentally infected group on day 4 p.i. Clinical scores of the experimentally infected beagles peaked on day 7 p.i. (Figure 1). And the dogs began with vomiting and three had bloody diarrhea. The infected dogs were given a supportive treatment with oral electrolytes on welfare grounds. But on day 9 p.i., the clinical signs in two dogs progressed to include severe vomiting, refusal of food and water, and profuse smelly, bloody diarrhea despite the supportive treatment, and therefore had to be euthanized. The remaining seven were treated by intravenous fluids, anti-nausea medications, and antibiotics to prevent secondary infections. On day 12 p.i., clinical signs of one dog were relieved, with the exception of mild diarrhea. The last six dogs showed no any signs of vomiting or diarrhea. The dogs in the control group remained healthy and had a normal body temperature (38.5 ± 0.2°C) throughout the experiment.

In the two humanely euthanized dogs, hemorrhagic enteritis of the small intestine was the predominant gross lesion observed from the pathology autopsy. There were no apparent gross lesions in other tissues (data not shown).

### Virus detection in rectal swabs and sera

All rectal swabs that were collected from all dogs at 1 day before infection were CPV negative. On day 4 p.i., some CPV-infected dogs (5/9) showed positive results in rectal swabs. All rectal specimens from CPV-infected dogs were positive after day 5 p.i. However on day 12 p.i., the number of positive results in rectal swabs decreased, and 2/7 rectal swab samples showed negative results.

Viral DNA was detected in the sera from six of nine infected dogs four days after inoculation and in all infected dogs five days after inoculation. But from day 8 p.i., no viral DNA was detected in any infected dogs. Further virus isolation demonstrated that CPV could be recovered from the dogs with positive viral DNA, but not from those with negative viral DNA (data not shown).

### Real-time PCR for quantitation of viral loads

**Figure 2:**
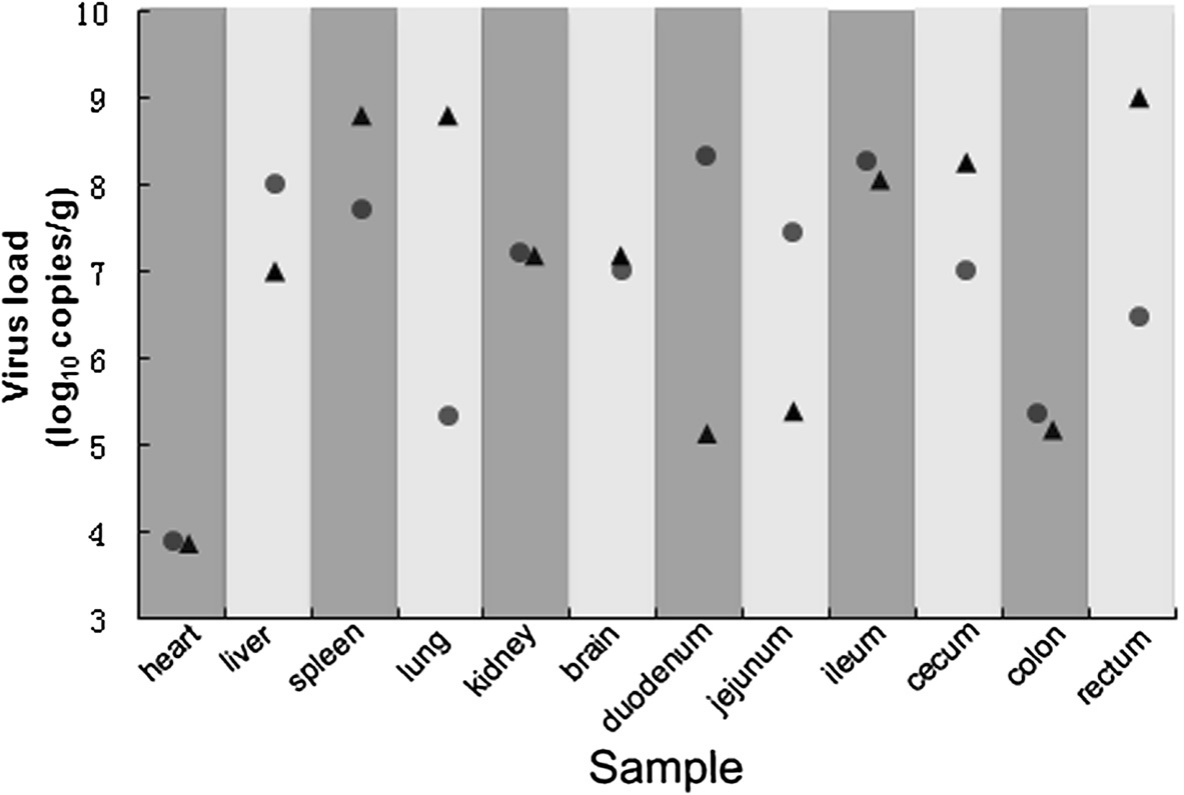
**Virus loads in the main organs of two humanely euthanized beagles infected experimentally CPV-JS2.** Every column represents virus load in an organ sample from one beagle. Viral loads are expressed as log_10_ DNA copy numbers per gram of sample. beagle No.4; beagle No.6.

Real-time PCR assays were carried to measure viral DNA loads in the main organs of two humanely euthanized dogs. All tested samples were positive and mean viral DNA loads were above 10^5^ copies/g, except for the heart (<10^4^). Interestingly, the virus load was high in the brain (>10^7^copies/g). There were some differences in viral DNA loads of affected tissues between the two dogs. Rectum was the tissue with highest viral DNA load (1.02 × 10^9^ copies/g) in beagle No.4, but duodenum was the highest tissue (2.22 × 10^8^ copies/g) in beagle No.6 (Figure 2).

### Virus isolation from the brain

In one from two brain samples processed was possible to isolate canine parvovirus in F81 cells. The presence of the virus in the cell cultures was confirmed by cytopathic effect (CPE), viral hemagglutination (HA) and PCR. Typical CPE of parvovirus, such as rounding and detachment, appeared in the cells after second passage. The highest HA titer of the virus was 1: 64 with porcine erythrocytes. A single DNA band of the expected size (583 bp), corresponding to the partial amplification of the VP2 gene, was observed by gel electrophoresis.

### Histopathology and immunohistochemistry

**Figure 3:**
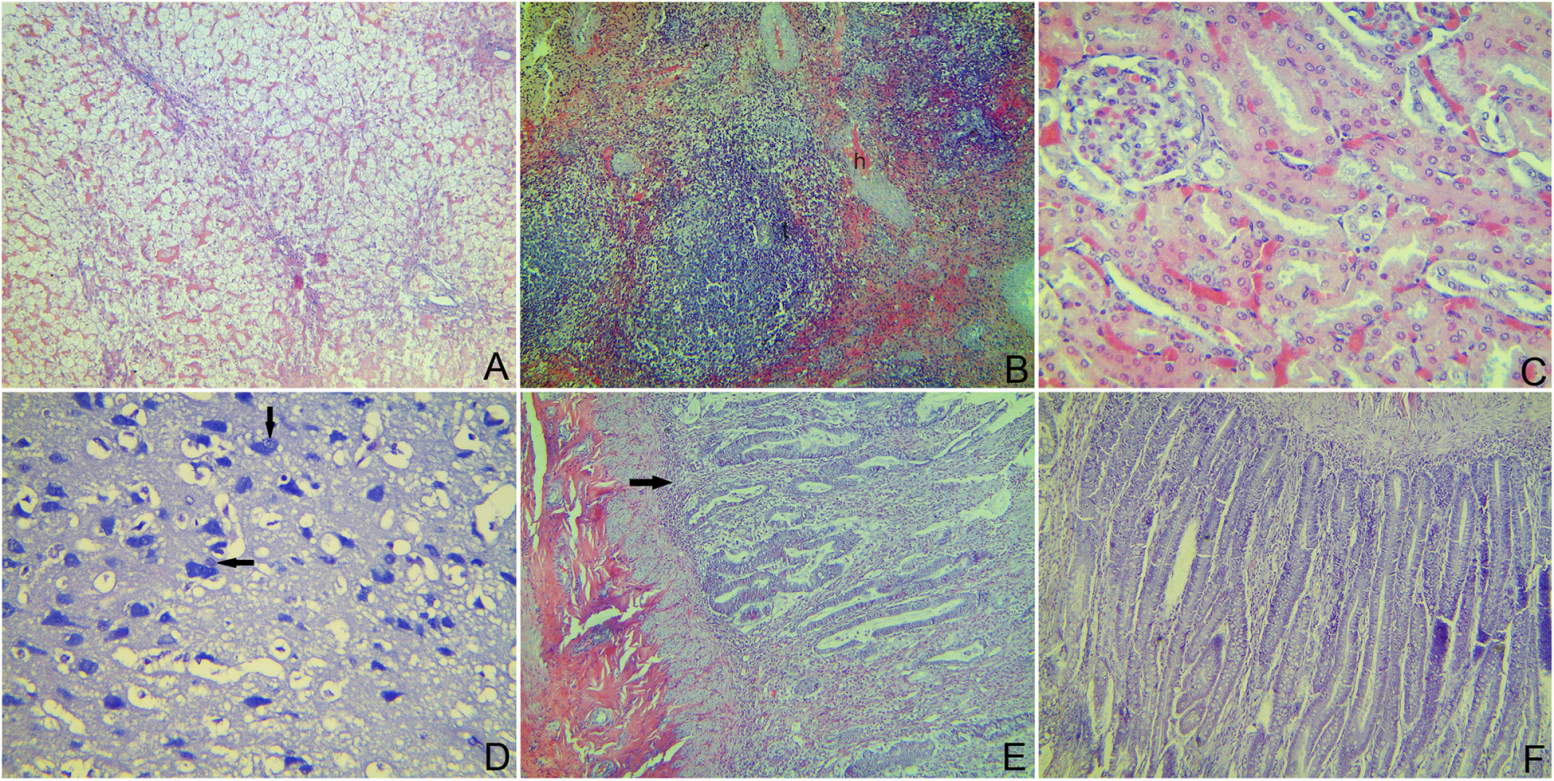
**Histopathologic appearances of H&E-stained liver, spleen, kidney, brain, duodenum and jejunum of two humanely euthanized beagles infected experimentally CPV-JS2. A**: liver, showing extensive degenerated hepatocytes, hyperemia of hepatic sinusoid; 100×. **B**: spleen, showing the significantly thickened periarterial lymphatic sheath (t), and the hyperemia in artery of spleen trabecular (h); 100×. **C**: kidney, showing endothelium and mesenchymal cell proliferation in glomerulus, and renal tubules epithelia degeneration and necrosis; 400×. **D**: brain, showing neuronophagia (arrow); 400×. **E**: duodenum, showing necrosis of mucous membrane (arrow) and cell proliferation in lamina propria; 100×. **F**: jejunum, showing necrotic epithelia; 100×.

On day 9 p.i., tissue samples from two humanely euthanized beagles were collected for histopathological examinations and immunohistochemistry (IHC) stain. The two dogs showed the similar histopathologic lesions and viral antigen staining in every specific tissue. There were typical histopathologic lesions in all tested tissues, except the heart and the lung. Extensive degenerated hepatocytes were swollen and rounded with vacuoles of varying sizes in the cytoplasm. Hepatic sinusoid was hyperemia with erythrocytes (Figure 3A). The number of lymphocytes in periarterial lymphatic sheath was significantly increased and this area was thickened (Figure 3B). Besides, the hyperemia in artery of spleen trabecula may be a suggestion for inflammation. In kidney, glomerulus was swollen and filled up Bowman’s capsule. Endothelium and mesenchymal cell in glomerulus showed proliferation. Epithelia of renal tubules underwent degeneration and necrosis (Figure 3C). Glial cells hyperplasia came with neuronophagia (a degenerative or dead neuron was engulfed by microgliocytes) (Figure 3D). Similar pathological lesions were observed in all segments of the small and large intestines. Disruption of the villous architecture was characterized by blunting and fusing of villi. Epithelia of mucosa underwent degeneration, necrosis and desquamation. These changes appeared to be more significant in the duodenum (Figure 3E) and the jejunum (Figure 3F).

**Figure 4:**
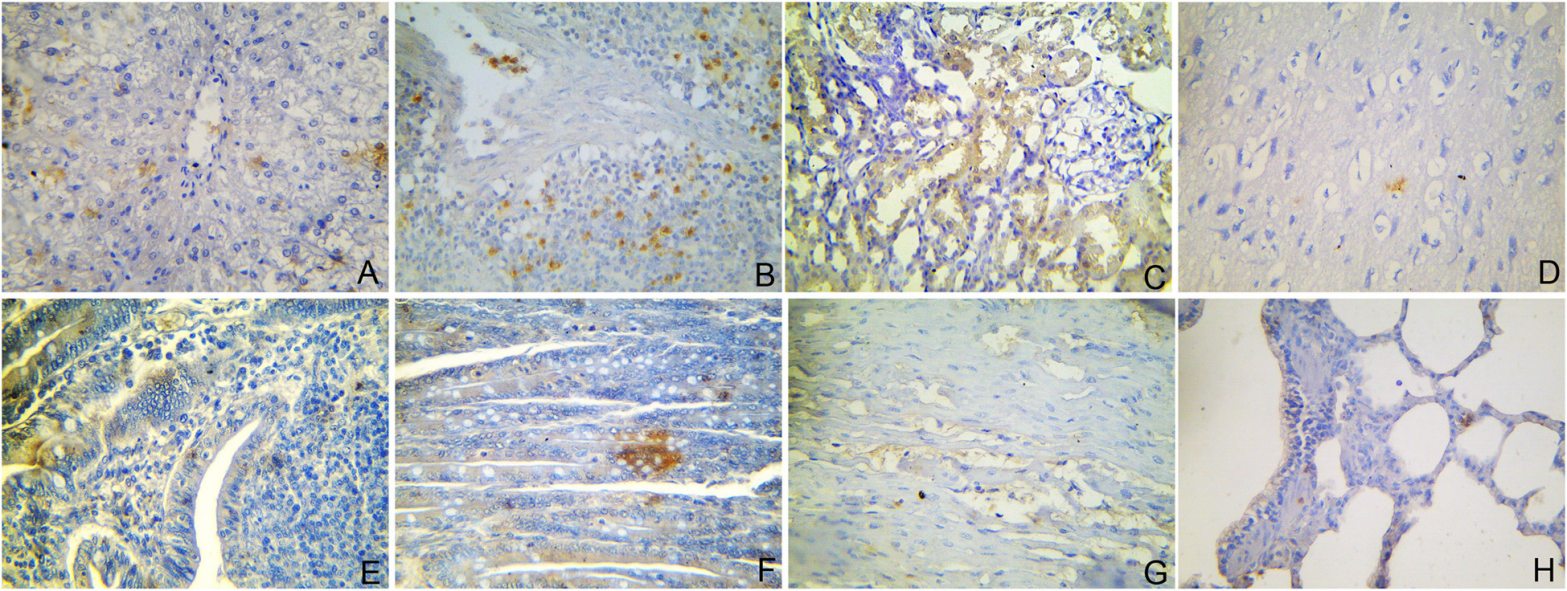
**Immunohistochemistry (IHC) detection of CPV antigen in collected tissues of two humanely euthanized beagles infected experimentally CPV-JS2. A**: liver, virus antigens in degenerated hepatocytes; 400×. **B**: spleen, virus antigens in marginal zone cells; 400×. **C**: kidney, virus antigens in necrotic renal epithelial cells; 400×. **D**: brain, virus antigens in necrotic nerve cells; 400×. **E**: duodenum, virus antigens in columnar epithelium of intestinal gland; 400×. **F**: jejunum, virus antigens in necrotic epithelium; 400×. **G**: heart, virus antigens in myocardial fibers; 400×. **H**: lung, virus antigens in bronchioles epithelium, 400×.

In the IHC staining, viral antigens were detected in the lesions of various organs. They were primarily found in degenerated hepatocytes (Figure 4A), the marginal zone of spleen (Figure 4B), necrotic renal epithelial cells in the kidney (Figure 4C), necrotic nerve cells in the cerebrum (Figure 4D), and necrotic epithelia of the duodenum (Figure 4E) and the jejunum (Figure 4F). CPV antigens were seen also in myocardial fibers of the heart (Figure 4G) and in bronchiolar epithelial cells and walls of atria of the lung (Figure 4H) although no apparent histologic lesions in these organs.

## Discussion

Point mutations in the VP2 protein have been associated with the CPV types. Sequence analysis can give ample information for CPV typing since the fragment amplified by conventional PCR encodes for at least one informative aa (residue 426) of the VP2 protein [23]. The change of the amino acid at position 426 can differentiate the CPV2a (Asn), CPV2b (Asp) and CPV2c (Glu) [24]. CPV has a worldwide distribution. In many countries in Europe, such as United Kingdom, Germany and Italy, CPV-2a has been overtaken by CPV-2b or CPV-2c. Type 2b and 2c isolates predominate in North America [11,12], whereas CPV-2c is more widespread in South America [13,14]. India has reported the prevalence of CPV-2a and CPV-2c [25]. This study, together with previous findings [15,26], indicated that CPV-2c has not been detected in China. The sequencing results of 31 CPV samples indicated that the CPV-2a variant is more popular than the CPV-2b variant in Nanjing, China. A similar epidemiological pattern has been reported in Brazil, where all circulating strains were characterized as CPV-2a [27]. Also, some reports showed that CPV-2a is the predominant variant in Asia [25,28,29] and Australia [30]. On the contrary, Zhang *et al*. [15] showed the high frequency of CPV-2b (21 out of 23) in China. But another research group [26] reported that three types of CPV, including CPV-2, CPV-2a, and CPV-2b, could be detected, and CPV-2a was predominant in China. These differences might be due to sample localities and periods. The former study focused on Jiangsu, Anhui and Zhejiang strains from 2006 to 2009 [15], while later samples were collected from Sichuan, Yunnan, Guizhou and Jilin Provinces from 1983 to 2008 [26]. Interestingly, only two strains of CPV-2a reported by Zhang *et al*. [15] were collected from Nanjing, further supporting the present study that CPV-2a is the prevalent subtype in Nanjing, China.

The deduced amino acid sequences from 31 VP2 gene products in this work revealed the variability at residues 427, 440, 445, 512 and 580. Interestingly, the greatest variability in the VP2 protein occurred (within the GH loop) at position 440. This finding is in accordance with the finding of Kang *et al*. [28]. It has been reported that a high level of substitution in this region has been associated with the evolution of antigenic variants in circulating parvovirus types [31-33].

In the present experiment, inoculation of CPV-2a induced significant clinical symptoms in all infected animals, and two of them had to be euthanized. It is not clear whether one CPV-2 variant has a greater infection or virulence over the others. The previous work showed that experimental infection of beagles with the original type 2 infrequently resulted in severe clinical disease like that seen in natural disease [34]. Meunier *et al*. [35] reported that the original type 2 caused clinical signs in about 30% of infected beagles. In comparison to the original type 2, the antigenic variants 2a and 2b have been reported to cause a more severe disease [36]. Moon *et al*. [37] found that the overall pathogenicity of the CPV-2a variants (CPV-2a-I and 2a-V) was severer compared to the CPV-2b variant. From the above works, pathogenetic potential of CPV seems to be relative to the types of variants. But recently, there are a few published studies that described CPV-2c infections. Decaro *et al*. [38] reported that all pups infected with CPV-2c displayed clinical signs of parvovirosis, but none showed either hemorrhagic diarrhea or vomiting and all recovered in a few days. The findings from Spibey *et al*. [19] showed that all six of the infected dogs with CPV-2c became severely ill, and three of them had to be euthanized. Therefore it is reasonable to think that the types of CPV variants may not determine their pathogeneticity. This speculation is supported by the investigation from Decaro *et al*. [39] that similar patterns of tissue distribution were observed in all the examined dogs irrespective of the antigenic variant causing the disease.

Parvovirus replication in dogs was mainly seen in highly mitotically active tissues, such as lymphoid tissue, bone marrow, or the epithelium of the gastrointestinal tract [40]. Despite the existence of several studies on the antigenic characterization and geographic distribution of type 2a and 2b CPVs in China, few data are available on the pathobiology of the CPV-2 variants. Our study showed that, the CPV-2a variant comprises 93.5% (29/31) of the field strains in Nanjing, China. All the experimentally infected beagles with CPV-2a resulted in clinical disease similar to that of natural disease (mucoid or bloody diarrhea, vomiting and etc.). Especially on day 9 p.i., two of nine had to be euthanized due to the poor prognosis even after treatment, suggesting that the Chinese CPV-2a isolate was life-threatening. Also, virus shedding from feces could be detected from day 4 p.i., and this could represent a source of viruses which could potentially infect other susceptible dogs.

CPV DNA was demonstrated in all the sampled tissues, showing a wide distribution of the virus in the infectious dogs. To demonstrate the presence of viral DNA is associated to effective replication and expression of viral proteins in the sampled organs, we identified CPV antigens using immunohistochemistry. All tested samples showed positive antigen staining. It suggests that the disease may lead to a generalized infection and cause viral replication and attack in other tissues other than the gastrointestinal tissues. The systematic infection was probably due to viral spread in the organism through the blood. This assumption can be supported by this finding that viremia was detected from day 4 p.i.

Among the tested samples, the viral DNA loads of the heart were lowest. And histological examination showed no significant lesions in this organ. Although CPV is known to be also able to replicate in cardiac cells and induce a fatal myocarditis, this form is rare in countries where vaccination of breeding dogs is common [41]. Interestingly, this virus appears to be capable of replicating in cerebral neurons in dogs. To further demonstrate this, we performed the virus isolation from brain samples and obtained positive result. To our knowledge, the parvovirus isolation from canine brain has never been reported. Schaudien et al. [42] also found that parvovirus antigen, as well as parvovirus DNA and mRNA, was detected within the brains of five Cretan Hound puppies suffering from a puppy shaker syndrome, further supporting the possibility of infection of the canine brain during systemic parvovirus infections. However, the findings are different from a precious report, wherein parvovirus antigen was not detectable in 40 canine brains of dogs with parvovirus enteritis [43]. The significantly different results might be due to viral strains, the host’s immunity, or the time point of infection.

The results pointed here may help to achieve a better understanding of the current status of CPV-2 infection in dogs from Nanjing, China. However, as to virulence of CPV-2a, since only one strain was examined, we can not determine whether CPV-2a circulating in Nanjing is more lethal or different in its tropism than others. More works will need to be done in the future study.

## Conclusions

The present study demonstrated that 2a is the main CPV type that circulated in Nanjing, China between the years 2009 and 2012, and the prevalent virus has been shown to be able to spread to all sampled tissues in dogs, including the brain. Further epidemiological surveillance and pathogenetic investigation of the new antigenic strains of CPV are needed for controlling parvovirus diseases in dogs.

## Methods

### Sampling and sample preparation

Rectal swab specimens (n = 70) were collected from the dogs diagnosed with CPV infection, 1 to 15 months, in 8 major animals hospitals located in Nanjing, the capital city of Jiangsu province in China, from years 2009 to 2012. Only the animal hospitals with the capability of performing the diagnostic studies were involved in this study. The diagnostic criteria for CPV infection were based on clinical signs, the examination of blood and antibody titration or antigen detection. Final diagnosis considered compatible with CPV enteritis included hemorrhagic gastroenteritis, gastroenteritis or acute onset of vomiting or diarrhea. The positive laboratory test results for CPV included leukopenia (a WBC of <5000 cells/μl) or a single CPV hemagglutination inhibition (HI) antibody titer ≥ 1: 5120 [44]. The samples were tested positive to CPV-2 by a CPV commercial diagnostic kit (Rapitest, South Korea).

The collected samples were emulsified in 1 ml sterile phosphate-buffered saline (PBS) and centrifuged at 10000 rpm for 10 min at 4. The supernatant was collected for PCR amplification.

### DNA extraction and PCR amplification

Hundred microlitres of the supernatant was used for template DNA preparation with a Qiagen viral DNA kit. About 0.1 to 1 μg of DNA was added per reaction. PCR amplification was carried out under standard conditions using VP2-F (5′-CAGGAAGATATCCAGAAGGA-3′) and VP2-R (5′-GGTGCTAGTTGATATGTAATAAACA-3′) [23]. Amplicons (583 bp) were visualized by GelSafe nucleic acid dye (10000 × solution; YuanPingHao-bio, Beijing, China) added to 2% agarose gels after electrophoresis was complete. Sterile water was used instead of the specimen as the negative control in every test. PCR products were purified with the Agarose Gel DNA Purification Kit (TaKaRa) and cloned into the pMD18-T vector (TaKaRa). Positive clones were selected and sequenced.

### Virus isolation

Fecal samples from three dogs which were positive by PCR were used for virus isolation. The procedure was carried out as described by Hirayama *et al*. [45]. Briefly, the sample was homogenized (10%, w/v) in PBS (pH 7.2) and subsequently clarified at 12000 rpm for 10 minutes. The clear supernatants were filtered using 0.22 μm membrane filter (Millipore). Then filtrates were treated with penicillin and streptomycin at 4 overnight and inoculated into F81 cells. The infected monolayers with CPE were harvested at 4 days post-infection by three cycles of alternative freezing and thawing. The virus supernatants were screened for the presence of virus by PCR using the same primer pair VP2-F/VP2-R. The CPV suspensions were titrated using 50% tissue culture infective dose (TCID50) assay [46]. Also, the samples were collected for morphological examination of viral particles by negative staining and electron microscope (Hitachi H-7650) [47].

### Sequence analysis

In order to ensure that one sample was selected for sequence analysis at each clinic each year, we used a sample proportion of 40% from 2010 to 2012. All the four samples from Animal Clinics of Nanjing Agricultural University in 2009 were selected because of limited data. Therefore, a total of 31 samples were sequenced. The sequences from this study were compared with the VP2 sequences of 5 reference strains of CPV (CPV-15, CPV-V120, CPV-39, CPV-Taichung and CPV-G7/97). Reference sequences were obtained from the National Center for Biotechnology Information (NCBI; http://www.ncbi.nlm.nih.gov). Comparisons of nucleotide and deduced amino acid sequences were made using DNASTAR software.

### Experimental infection

Twelve healthy beagles aged 7–8 weeks old were obtained from unvaccinated unexposed bitches in Nanjing Medical University Experimental Animal Center, and therefore devoid of maternally derived antibodies against canine parvovirus. They were housed in a BSL-2 isolation facility under standard husbandry conditions. The dogs were kept 1 week for acclimation before experiment. Food and water were provided ad libitum. All the experiments were performed in a BSL-2 isolation facility. Dogs were confirmed to be negative for CPV using HI assays as described by Jeoung *et al*. [48]. All animal experiments complied with the guidelines of Animal Welfare Council of China. The protocol was approved by the Animal Ethics Committee of Nanjing Agricultural University. All efforts were made to minimize suffering. Dogs used in this study were housed and cared for in accordance with the Guide for the Care and Use of Laboratory Animals (eighth edition). During the experiment, the animal’s condition was assessed by a responsible veterinarian.

Nine beagles were randomly chosen and assigned into the experimentally infected group. A 1 ml aliquot of virus stock (10^5.86^ TCID_50_/ml) was used to inoculate each of nine beagles oronasally. The other three beagles were inoculated with 1 ml sterile PBS each as control. To avoid contact transmission, each dog is raised alone in the cage.

### Clinical observations

Clinical observations (e.g. body weight, body temperature, and the presence of diarrhea) of each animal were monitored for 12 days post infection (p.i.) or until death using a record sheet. Some clinical signs were assigned scores to evaluate the severity of the disease as described by Moon *et al*. [37], with some modifications, and given in Table 2. The average clinical scores were calculated based on the daily score. The number of white blood cell (WBC) was determined using an automated blood cell counter (ABX MicRos 60, France).

**Table 2:** Assessment of clinical scores

**Body temperature (°C)**	**WBC count (×10**^**3**^ **μl**^**-1**^**)**	**Diarrhea**	**Loss of appetite**	**Vomiting**	**Depression**
0 = 37.7-39.4	1 = 5.9-4.0	1 = Mucoid	0 = Absent	1 = Mild	0 = Absent
1 = 39.5-39.9	2 = 3.9-2.0	2 = Fluid	1 = Present	2 = Moderate	1 = Present
2 = 40–40.4	3 = <2.0	3 = Bloody		3 = Severe	
3 = ≥40.5					

### Sample collection

Rectal swabs were collected individually to detect virus shedding from days 1 to 12 p.i. The swab was removed from anus and the tip of the swab was immersed carefully in an Eppendorf tube that contained 1 ml of virus transport medium (phosphate-buffered saline containing 10000 U/ml penicillin, 10000 μg/ml streptomycin and 250 μg/ml gentamycin). Also, serum samples from days 1 to 12 p.i. were collected for detection of CPV. Two beagles which were humanely euthanized on days 9 p.i. were necropsied and sampled. Heart, liver, spleen, lung, kidney, brain, duodenum, jejunum, ileum, cecum, colon and rectum were collected for virus detection and histopathological examinations.

### Virus detection and quantitative PCR

PCR amplification for the conserved region of VP2 gene was carried out to detect serum and fecal samples using the same primer pair VP2-F/VP2-R. Quantitative assays were carried to measure viral DNA loads in the main organs as described by Decaro *et al*. [49]. Real-time PCR was run in an ABI 7300 Real Time PCR System

### Histopathologic examination and immunohistochemistry

Tissue samples from two humanely euthanized beagles were fixed by submersion in Bouin’s solution and embedded in paraffin wax. Serial 4-μm sections were prepared for hematoxylin and eosin (H&E) staining and IHC. For the IHC assay, tissue sections were prepared on slides and stained using an immunoperoxidase test. Briefly, a 1:1000 dilution of rabbit-derived polyclonal antibody (produced in our laboratory using the purified CPV described in this study) was applied as the primary antibody, followed by the application of biotinylated goat anti-rabbit IgG as secondary antibody (DingGuo, Beijing, China), which was then detected by horseradish peroxidase (HRP)-conjugated streptavidin (DingGuo, Beijing, China). An enhanced HRP-DAB chromogenic substrate kit (TianGen, Beijing, China) was used for chromogenic detection following the manufacturer’s instructions, and then slides were counterstained with hematoxylin.

## Competing interests

The authors have no conflicts of interest to declare.

## Authors’ contributions

Conceived and designed the experiments: YBZ YL XJZ CPL JFH. Performed the experiments: YBZ YL XJZ. Analyzed the data: YBZ JFH. Contributed reagents/materials/analysis tools: CPL JFH. Wrote the paper: YBZ YL XJZ CPL JFH. All authors read and approved the final manuscript.
